# Conservative Rehabilitation Program for Osteochondroma of the Scapula: A Case Report

**DOI:** 10.7759/cureus.58293

**Published:** 2024-04-15

**Authors:** Ghanishtha C Burile, Swapnil U Ramteke

**Affiliations:** 1 Sports Physiotherapy, Ravi Nair Physiotherapy College, Datta Meghe Institute of Higher Education and Research, Wardha, IND

**Keywords:** strengthening, inverted j-shaped taping, scapular stabilization exercises, flat bone, benign neoplasms, pseudo winging, metaphysis, cartilage-capped outgrowths, osteochondroma

## Abstract

One of the most frequent cartilage-capped outgrowths that develop beneath the periosteum due to cartilage ossification is osteochondroma. The second decade of life is noted as the most prevalent age of presentation. This case report looks at an uncommon osteochondroma presentation in a 20-year-old female with swelling along the right inferomedial border of the scapula. The patient presented with complaints of difficulty in daily activities and exhibited altered posture, decreased range of motion (ROM), muscle weakness, and altered shoulder function. The clinical assessment highlighted restricted shoulder and cervical ROM and muscle weakness in the trapezius, rhomboids, serratus anterior, and other surrounding muscles. Magnetic resonance imaging revealed an inferomedial bony outgrowth indicative of osteochondroma. A comprehensive physiotherapy intervention protocol for eight weeks was designed to alleviate pain, improve mobility, restore ROM, strengthen weakened muscles, correct posture, and enhance functions that were restricted. The protocol encompassed various techniques, such as muscle energy techniques (MET), proprioceptive neuromuscular facilitation (PNF), cold therapy, stretching, scapular mobilization, resistance exercises with TheraBand, postural correction exercises, ergonomic adjustments, scapular stabilization exercises, and 'J'-taping to aid in muscle activation and address rounded shoulder posture. Outcome measures for cervical and shoulder ROM and strength were measured to note the progression after rehabilitation. The case report emphasizes the importance of a tailored physiotherapy rehabilitation protocol in managing osteochondroma-related symptoms, showing the potential benefits of multifaceted interventions in alleviating pain, improving function, and boosting the quality of life for individuals with similar presentations.

## Introduction

Osteochondroma is a common condition in which there are benign osseous surface lesions that mostly arise as a result of the metaphysis of the long bone, which makes up 20-50% of benign osseous tumors. When flat bones (pelvis, scapula, and spine) are involved, medullary continuity is less evident on radiographs, and cross-sectional imaging is often required to characterize definitively [1]. Osteochondromas are usually asymptomatic, but when they are bigger, they can produce symptoms such as bursa formation, mechanical pressure, neurovascular impingement, fractured bony stalks, and rarely malignant transformation of the cartilage cap [2]. Osteochondroma is usually painless, but the most common complaints in cases of scapular osteochondroma resulting from mass effect include pain, limited range of motion, impingement mechanism, nerve compression, and friction-related bursitis [3]. Flat bones are less likely to be affected by osteochondromas. The collection of tumors is also called multiple osteochondromatosis. Osteochondromas can occasionally develop into malignant lesions [4]. Osteochondromas have been documented in patients with scapula-affecting tumors or exostosin 1 (EXT1) mutations [5]. Scapular osteochondroma manifests as symptoms either directly from pressure on the surrounding anatomic structures or indirectly through reactive bursitis [6]. Osteochondroma rarely occurs in the scapula, accounting for 4.6% of all bone tumors [7]. Osteochondroma develops in tandem with bone development until physis closes [8].

These scapular tumors may present with winging of the scapula [9]. A common tumor associated with pseudo-winging is osteochondroma situated on the ventral surface of the scapula [10]. True winging, which results from neuromuscular conditions such as long thoracic nerve palsy, and pseudo-winging, which is caused by osseous and soft-tissue masses over the scapula and thoracic cage, are the two types of primary winging [11]. The classic etiopathology of scapular winging was injuries to the spinal accessory or long thoracic nerves, resulting in trapezius and serratus anterior palsy, respectively [12]. An extremely uncommon location for solitary osteochondroma is acromegaly, which might result in pressure complaints over nearby tissues such as the axillary nerve and the circumflex humeral arteries [13]. Some articles on osteochondroma of the scapula showed that many lesions are situated along the scapular equator, while the others that originate from the inferior border of the scapula tend to be bigger due to limited space [14]. To manage the symptoms of patients, there are some conservative methods, such as immobilization, anti-inflammatory medications, and local anaesthetic injections along with physiotherapy [15]. If the patient complains of pain or functional limitation due to neurovascular compression, leading to limitation of joint movement, then surgical removal of the tumor is recommended [16].

## Case presentation

Patient information

We are addressing the rare case of a 20-year-old female who complained of pain and swelling over the inferno medial border of the scapula on the right side for the last three months. The patient acknowledged a pea-sized swelling two months back. The pain was gradual in onset, dull aching, and non-radiating. She also complains of difficulty in performing activities of daily living such as carrying a heavy bag on her shoulders, difficulty in driving, difficulty in sitting for long hours, and difficulty in writing. The pain was relieved at rest and with medications. At the time of admission, an assessment of strength, range of motion (ROM), and posture was done, which revealed reduced strength and ROM of both cervical and shoulder joints. In the postural examination, the right-sided shoulder was more elevated as compared to the left one.

Postural examination

**Figure 1:**
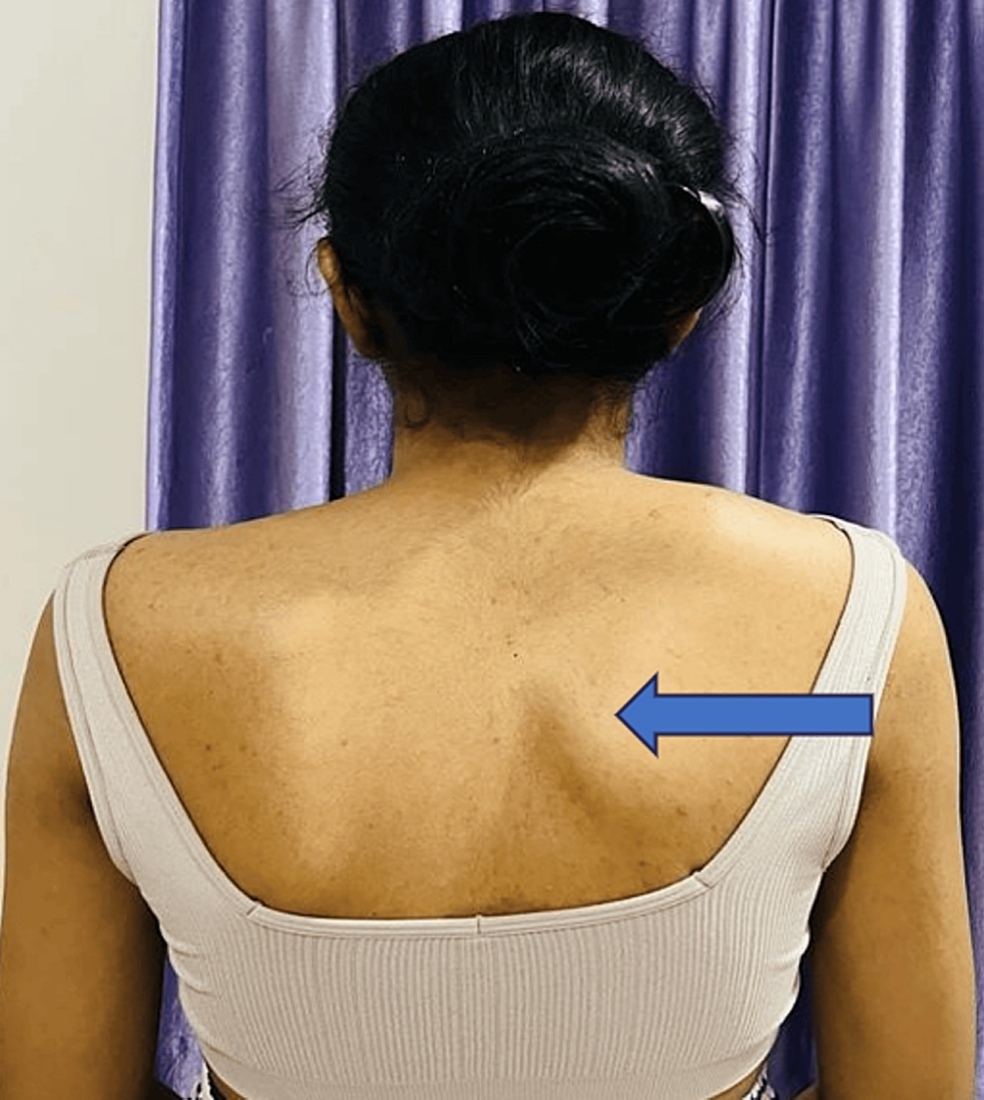
Posterior view of the pseudo winging of the scapula on the right side (blue arrow)

Figure 1 presents the postural examination in the posterior view that indicates the pseudo winging of the scapula on the right side (indicated with a blue arrow).

**Figure 2:**
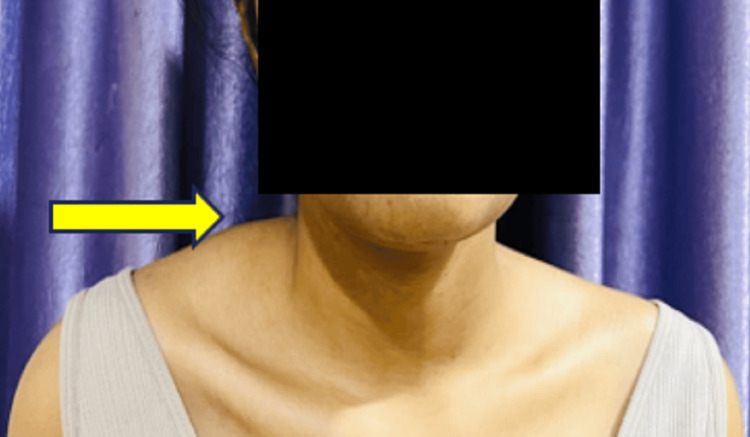
Anterior view of the right side of the shoulder, which is more elevated compared to the left one (yellow arrow)

Figure 2 presents the anterior view of the right side of the shoulder, which is more elevated compared to the left one (indicated with a yellow arrow).

**Figure 3:**
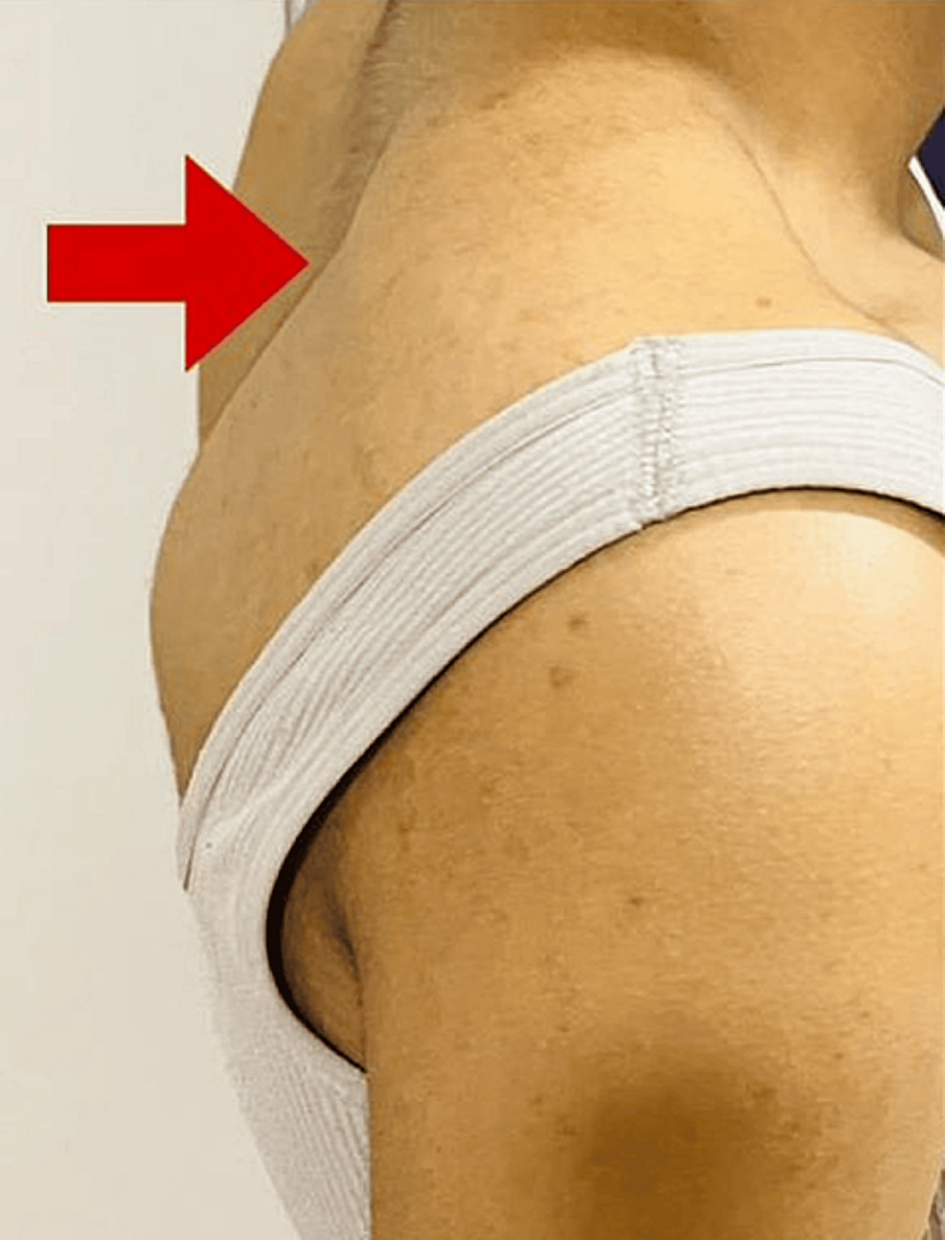
Lateral view of the medial border of the scapula, which is more prominent

Figure 3 presents the lateral view of the medial border of the scapula, which is more prominent (red arrow).

Outcome measures

Outcome measures used in the above study are shoulder joint, cervical ROM, and manual muscle testing. A comparison of shoulder and cervical ROM before and after rehabilitation is presented in Table 1.

**Table 1:** Comparison of the ROM of the shoulder and cervical joint before and after rehabilitation ROM: range of motion

	Before treatment		After treatment	
Shoulder joint	Active	Passive	Active	Passive
Flexion	0-70^o^	0-75^o^	0-160^o^	0-170^o^
Extension	0-30^o^	0-35^o^	0-55^o^	0-60^o^
Abduction	0-70^o^	0-70^o^	0-170^o^	0-180^o^
Cervical				
Flexion	0-30^o^	0-35^o^	0-40^o^	0-45^o^
Extension	0-30^o^	0-35^o^	0-40^o^	0-45^o^
Lateral flexion	0-40^o^	0-40^o^	0-45^o^	0-45^o^
Rotation	0-50^o^	0-55^o^	0-60^o^	0-60^o^

Manual muscle testing according to the Oxford Grading Scale is presented in Table 2.

**Table 2:** Manual muscle testing according to the Oxford Scale 2+: full range of motion in gravity eliminated plane, breaks upon minimum resistance; 3: full range of motion against gravity with no resistance; 3+: full range of motion against gravity, breaks upon minimum resistance; 4-: full range of motion against gravity, with less than moderate but more than minimum resistance

	Before treatment		After treatment	
Joints	Right side (affected)	Left side	Right side (affected)	Left side
Shoulder flexors	2+	2+	3	3+
Shoulder extensors	2+	2+	3	3+
Shoulder abductors	2+	2+	3	4-
Cervical flexors	2+	2+	3	4-
Cervical extensors	2+	2+	3	4-
Cervical lateral flexion	2+	2+	3	4-
Cervical rotation	2+	2+	3	4-

Investigations

**Figure 4:**
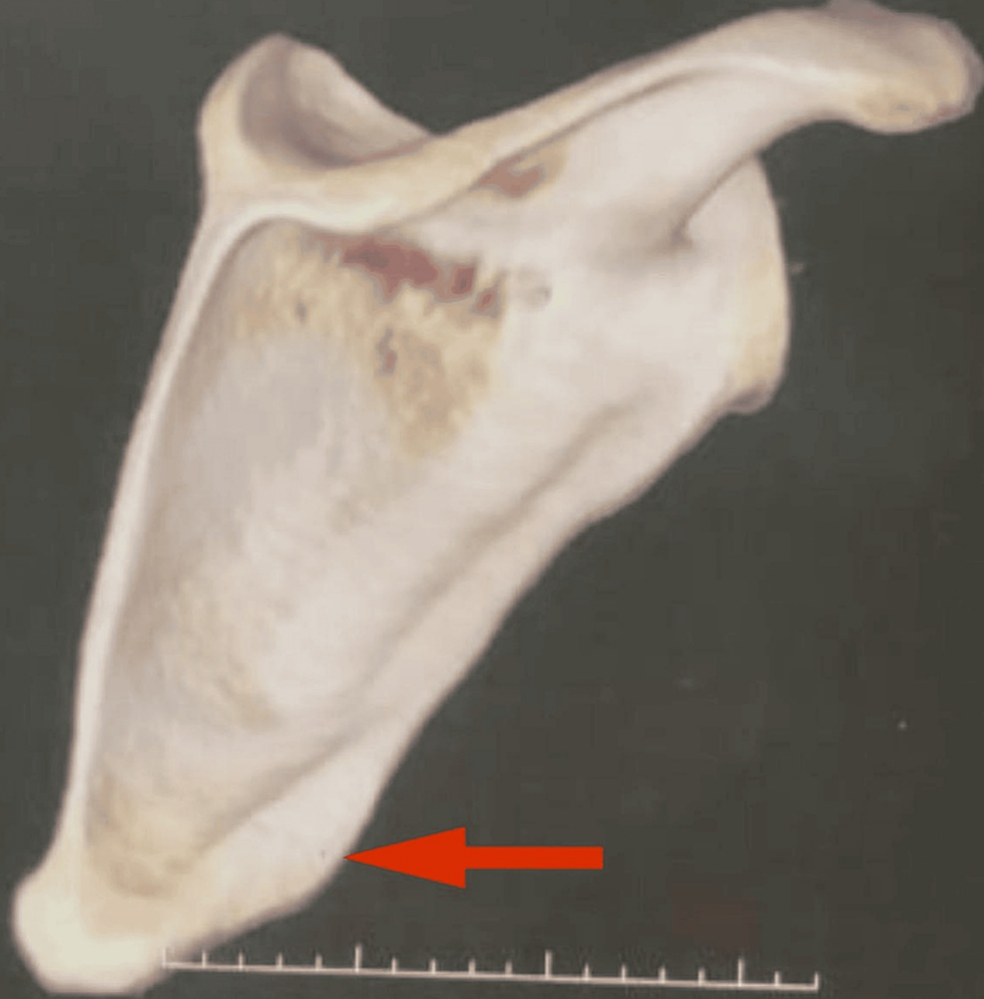
MRI of the right shoulder revealed an inferomedial border exophytic bony outgrowth measuring 1.7 cm in length and 6 mm in thickness on the right side of the scapula (red arrow)

MRI of the right shoulder revealed an inferomedial border exophytic bony outgrowth measuring 1.7 cm in length and 6 mm in thickness on the right side of the scapula (indicated with a red arrow in Figure 4).

Physiotherapy intervention protocol

As a tailored rehabilitation protocol to alleviate pain, enhance mobility, and improve function, this protocol employs a multifaceted approach integrating targeted interventions for eight weeks (Table 3).

**Table 3:** Physiotherapy rehabilitation protocol PNF: proprioceptive neuromuscular facilitation; ROM: range of motion [17]

Problem list	Goals	Interventions	Repetitions
Patient complains of pain and discomfort	To alleviate pain and make the patient comfortable	Muscle energy technique (MET) for upper back muscles	5 repetitions, 3 sets/session, 1 session/day
Reduced mobility limited range of motion	To improve mobility and range of motion at the cervical and shoulder joint	PNF D2 flexion-extension pattern with hold relax technique for cervical spine	10 repetitions × 2 sets
Swelling over scapula	To decrease swelling	Cold therapy	20 minutes initially 3 times and then progress to reduce depending upon symptoms of the patient
Limited range of motion	To restore full ROM	Passive and active stretching at cervical and shoulder joint muscles, scapular mobilization	10 repetitions × 2 sets twice a day
Muscle weakness	To improve muscle strength in the trapezius, rhomboid, and other surrounding muscles	Resistance exercises, progressive muscle strengthening of the trapezius, serratus anterior, rhomboids, deltoid, teres major and minor with the help of TheraBand initially with yellow and progress to red, blue.	10 repetitions × 2 sets once a day
Altered posture	Correct posture abnormalities	Postural correction exercises and ergonomic adjustments. As the patient has protracted shoulders inverted ‘J’taping can be used for muscle activation as it helps in correcting rounded shoulder posture when the scapula is protracted	Once a day
Dysfunction at shoulder joint	Improve functioning at the shoulder joint	Scapular stabilization exercises T’s and Y’s	10 repetitions × 3 times a day
Increase tension in the neck, shoulder, and upper back	Relieve the muscle tension in the neck, shoulder, and upper back	Scapular bracing	Should be used while working for a long time

**Figure 5:**
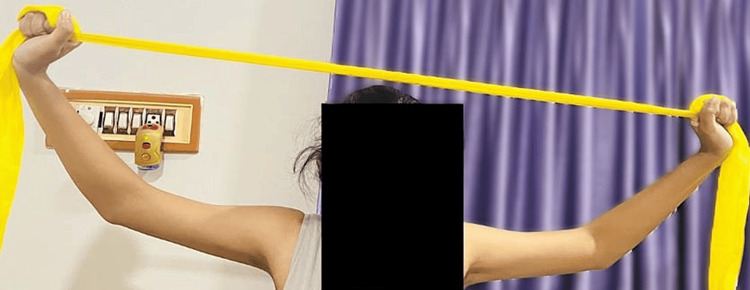
Patient performing strengthening exercises with the help of TheraBand (yellow) for the upper trapezius and rhomboids

Figure 5 shows the patient performing strengthening exercises with the help of TheraBand (yellow) for the upper trapezius and rhomboids.

**Figure 6:**
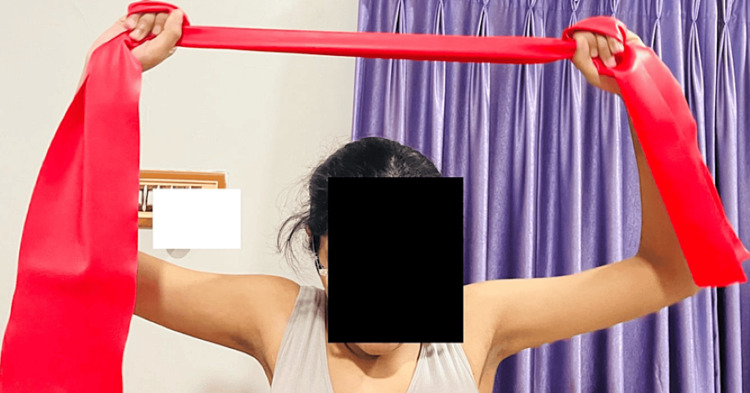
Patient performing strengthening exercises with the help of TheraBand (red) for the upper trapezius and rhomboids

Figure 6 shows the patient performing strengthening exercises with the help of red TheraBand for the upper trapezius and rhomboids.

**Figure 7:**
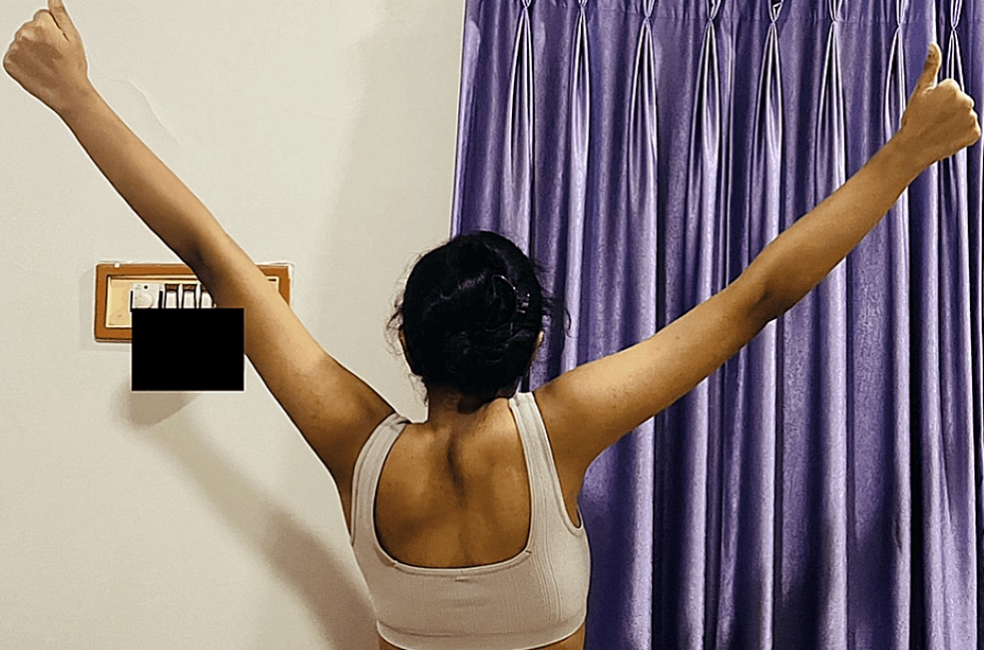
Scapular ‘Y’ stabilization exercise

Figure 7 shows the patient performing a scapular 'Y' stabilization exercise to improve shoulder joint function by stabilizing the scapula and restoring its position, direction, muscle movement control, and movement pattern.

## Discussion

Osteochondromas are benign tumors made of spongy bone covered by a cartilaginous cap. In this case, a 20-year female with scapular osteochondroma presented with complaints of swelling along the inferomedial border of the right scapula. This unique manifestation caused difficulty in daily activities, altered posture, decreased ROM, muscle weakness, and altered shoulder function. The clinical assessment highlighted restricted shoulder mobility and muscle weakness in several associated muscles. A study reported on a female patient who had a case of "adventitious bursa" related to a scapular exostosis. Any bone can produce osteochondroma, primarily seen in the metaphyseal area of long and flat bones, and stops growing after skeletal maturity [18]. The study researched by Abat on the snapping scapula found a strong relationship to tumors of the scapulothoracic region. The snapping scapula syndrome is a grating sensation located in the scapulothoracic region that appears with movement. This sign is occasionally related to tumors; therefore, it is crucial to understand [19,20]. Additionally, it is very important to plan the rehabilitation protocol applied, which was comprehensive and spanned over eight weeks. The patient's progress was monitored through various outcome measures, including shoulder joint and cervical ROM, and manual muscle testing.

The physiotherapy protocol involves an array of techniques such as muscle energy techniques (MET) used for relaxation and lengthening of the muscles and improving the ROM of the cervical spine; the proprioceptive neuromuscular facilitation (PNF) hold and relax technique used for reducing pain and increasing strength of the upper limb; cold therapy for relaxing the muscles and reducing swelling at the upper back patient, which was given two to three days for 8-10 minutes daily; stretching of tight muscles, which is important as it helps improve flexibility to maintain the range of motion of joints; resistance exercises with TheraBand for strengthening of weak muscles such as trapezius, rhomboids, serratus anterior, and other scapular muscles; postural correction exercises for maintaining good posture and for avoiding early fatigue; ergonomic adjustments using a brace; scapular stabilization exercises (Y and T) to improve movement pattern; and the use of 'J' taping to aid muscle activation and address rounded shoulder posture. The PNF technique, specifically the hold-relax method, was used to enhance the strength of the muscles surrounding the cervical spine while improving the quality of movement in the cervical spine. All the interventions were used to reduce the symptoms of the patient and improve functional recovery to improve quality of life.

## Conclusions

This case report underscores the importance of a targeted and multifaceted physiotherapy rehabilitation protocol in treating patients with scapular osteochondroma. It provides valuable insights into the efficacy of various interventions. It highlights the significant improvements achievable in alleviating pain, enhancing function, and the quality of life for individuals presenting with similar osteochondroma-related symptoms. Continued research and tailored rehabilitation approaches are crucial for optimizing the management of such rare and complex musculoskeletal conditions.
